# Evolution of gender research in the social sciences in post-Soviet countries: a bibliometric analysis

**DOI:** 10.1007/s11192-022-04619-9

**Published:** 2023-02-01

**Authors:** Zumrad Kataeva, Naureen Durrani, Zhanna Izekenova, Aray Rakhimzhanova

**Affiliations:** Graduate School of Education, Nazarbayev University, Astana, Kazakhstan

**Keywords:** Gender, Social science, Post-Soviet, Bibliometric analysis

## Abstract

While interest in mapping the patterns of publication and research in post-Soviet countries has been growing steadily, there is a gap in knowledge about how gender is featured in post-Soviet research and publications. Using a descriptive bibliometric approach and metadata extracted from 2822 publications indexed in the Web of Science Core Collection database for 1993–2021, this study seeks to understand the evolution of gender research in post-Soviet countries. The analysis revealed a notable increase in gender research and publications since the breakup of the Soviet Union, particularly between 2017 and 2021; however, the contribution of the post-Soviet countries to international research on gender remains insignificant. Russia, Estonia and Ukraine are at the forefront of gender research among post-Soviet states, while Caucasus and Central Asian countries, except Kazakhstan and Kyrgyzstan, contribute to the lower degree. Although research collaboration among post-Soviet researchers is increasing, researchers tend to mainly collaborate internally with authors from the same countries and institutions, and very few authors collaborate across post-Soviet states or internationally. The topics of publications in post-Soviet gender research incorporate different subjects, with most articles published within psychology and behavioral sciences, while gender research in sociological and political economy disciplines is still evolving.

## Introduction

For the past few decades, gender studies have become integral to various academic disciplines. However, the history of the development of the field is relatively short. Before the 1970s, the social sciences largely ignored gender, focusing primarily on men and politics while representing women in traditional ways as mothers and wives. The second wave of feminism in the late 1960s sparked the emergence of women’s studies throughout the West (Dahlerup, 2015; Thamarasseri, 2020; Woodward & Woodward, 2015). Feminist academics sought to understand gender relationships and the association of gender with inequality, oppression, and dominance (Curthoys, 2014). The rise of feminism and the women’s movement led to the development of centers and departments of women’s studies that strived to build empirical evidence and academic foundations for placing women on the political agenda and discussions over the role of women in the larger society began to emerge (Curthoys, 2014; Dahlerup, 2015).

Thamarasseri (2020) argues that during the early developments, "the primary concern of the field was incorporating gender into existing theoretical and critical lenses because women’s voices were underrepresented in the academies" (p.24).  Later, "the intellectual efforts evolved, expanding the theories and essential practices to include intersections of power, inequality, and privilege along the lines of sexuality, race, ethnicity, socioeconomic status, age, religion, physical embodiment, and other social markers" (Thamarasseri, 2020, p.24), as well as growing intellectual work in psychology, postcolonial, poststructuralist, sexuality, queer studies, and LGBTQ studies (Cleuziou & Direnberger, 2016, Williams & Round, 2008). As a result, the academic world began to refer to the discipline in which gender and gender relationships are considered central as gender studies rather than women’s studies, analyzing gender as a category (Curthoys, 2014; Woodward & Woodward, 2015). Since then, the interest in gender as an interdisciplinary field has become one of the most growing academic fields. For instance, interest in gender and gender issues led to approximately 70 research interest areas in the Australian Sociological Association in the past decade (Curthoys, 2014).

Moreover, the growing number of publications considered the issues of gender in politics, academia, economy, business, education and STEM subjects utilizing various theories and methodologies. Today, there are several interdisciplinary journals in gender studies, such as the International Journal of Feminist Studies and Women’s Studies International Forum, as well as journals within specific disciplines, such as Politics & Gender; Journal of Women, Politics & Policy; Feminist Economics; Gender & Society and Gender and Education or area study journals such as Journal of Middle East Women’s Studies (Thamarasseri, 2020). In addition, published research has focused on different gender concerns such as the reproduction of gender through education (Dunne, 2007; Durrani & Halai, 2018; Durrani et al., 2022), the gender representation of teachers and the perceived impact of the feminization of the profession on the outcomes of girls and boys (Apple, 2018; Eate et al., 2017; Humphreys et al., 2020), the symbolic significance of gender to nation building in newly independent states (Crossouard & Dunne, 2021; Dunne et al., 2020; Durrani & Halai, 2020), gender discrimination in academic promotions (Marini & Meschitti, 2018; Subbaye & Vithal, 2017), gender differences in performances of top-cited scientists (Chan & Torgler, 2020; Van den Besselaar & Sandström, 2016), and gender gaps in international research collaboration (Aksnes et al., 2019; Kwiek & Roszka, 2021), medical research (Merone et al., 2021; Sebo et al., 2021), STEM education and careers (Almukhambetova & Kuzhabekova, 2020; Kataeva, 2022) and leadership positions in educational institutions (Kataeva & DeYoung, 2017; Kuzhabekova & Almukhambetova, 2017).

While the development of gender studies was growing in Western countries, the concept of gender and gender research in the Soviet Union has taken another turn aligning the latter with the Marxist-Leninist ideology (Silova & Magno, 2004; Usha, 2005). In the first decades of Soviet rule, the Soviet ideology based on Marx’s writings was mainly concerned with class differences rather than gender differences and attributed gender inequality to economic relationships. Marxists argued that gender inequality exists in capitalism-born societies and will be resolved in socialist based relationships (Posadskaya et al., 1989). Thus, the essence of a Marxist socialist understanding included providing equal social status for women and men and their full participation in social and economic production. The Soviet policies aimed to bring women into the labor force by providing broad access to education and social services; however, in private life, the traditional arrangements of gender behavior continued to lead gender relations (Kataeva & DeYoung, 2017). Although the Soviet government provided a wide range of support, including nurseries, kindergartens, and after-school programs, which allowed women to combine their productive and reproductive roles, the government still controlled gender relationships through laws and policies such as abortion and family laws putting in place a ‘gender order” (Pushkareva & Bolshakova, 2020) and reinforcing the patriarchal relationships creating a “Soviet patriarchy” (Pushkareva & Bolshakova, 2020), the “Soviet paradox” of high literacy and labour force participation rates co-existing with large families and relatively untransformed domestic divisions of labor (Kandiyoti, 2007) or the “gender paradox”, the existence of equal educational participation alongside multisectoral gaps at the expense of women (Durrani et al., 2022).

Meanwhile, although Soviet science was an essential part of Soviet development, it was highly centralized and controlled by the government (Johnson, 2008). In particular, social sciences and humanities were considered parts of indoctrination and propaganda and have often been criticized for their highly politicized environment. The research was formally guided by behavioral psychology and Marxist positivist epistemology in quantitative statistical analyses and surveys (Shamatov et al., 2010). As the development of research could result in conflicting ideas with Soviet ideology, the government often limited the progress of the social sciences. Moreover, even if publications were not censored, they were shaped by ideological imposition and often represented existing political positioning rather than a conclusion based on empirical work (Shamatov et al., 2010). Because the Soviet government proclaimed gender equality as achieved by referring to statistics and econometrics analysis rather than social, cultural, and political measures, gender research and gender studies were largely not part of the social science disciplines until the late 1970s and 1980s.

The breakthrough in gender discussion occurred in 1989 when the issue of the periodic journal Kommunist published the article “How we solve the women’s issue” by Posadskaya et al. (1989). The authors raised a question about the division of labor between men and women in the Soviet Union emerging from social nature and social inequality. Although they did not mention the word ‘gender’, they criticized the patriarchal stereotypes that reinforce the naturalness and the inevitability of such disparities and outlined the ‘new’ approach to gender relations and gender approach. In addition, the traditions of recognising the role of women in the public and political spheres, as well as feminist ideas and feminist organizations led to the opening of the Laboratory of Gender Studies at the Institute for Socio-Economic Problems of Population (ISEPN) under the Russian Academy of Sciences in 1990 (Khotkina, 2020).

When the USSR collapsed, the abolition of censorship and the active participation of women in political and public life essentially affected the academic community throughout the former Soviet space. Moreover, the theories of feminism and gender new to these countries started to penetrate social and human sciences (Chatterjee & Petrone, 2019). Therefore, the 1990s is considered a “gender decade” because theoretical comprehension and, most importantly, public articulation of the problem of sexes existing in culture and public life occurred during this period (Fodor, 2002; Usha, 2005; Zimmerman, 2009). In this respect, transnational feminism and international funds supporting gender research played an essential role in raising awareness about gender issues (Chatterjee & Petrone, 2019; Zdravomyslova, 2009).

After the collapse of the Soviet Union in 1991, women scientists developed a new direction of social knowledge—gender studies, simultaneously participating in democratic changes in the territory of the former Soviet Union. The object of criticism was the ‘Soviet paradox’ or ‘gender paradox’, where the policies on families, allowances, public education, and kindergarten and child care resulted in a ‘double burden’ for women torn between work and home. At the same time, the changes that were taking place throughout the post-Soviet space opened sociopolitical activism, creating vast opportunities for political participation of previously marginalized actors (Ishkanian, 2005; Silova & Magno, 2004). The transformation process also allowed women to address their inclusion in the democratization process, engage in formal or informal politics, and demand that their particular issues be incorporated into the political system. Consequently, the collapse of the state control over political, economic, and social structures following the dismantling of the former socialist bloc has uncovered troublesome shortcomings of the legacy of socialist gender equality in the region (DeYoung & Constantine, 2009; Kataeva & DeYoung, 2017; Silova & Magno, 2004). The transition period to a neo-liberal economy was also characterized by an increased poverty rate that mostly affected women due to the withdrawal of governmental support, prior advantages and certain financial benefits that were present during the Soviet time.

The debates on the concepts of gender and gender studies in post-Soviet space took place around the discourses of women’s social status rather than women’s identity (Zdravomyslova, 2015; Zherebkina, 2003). For example, Zdravomyslova (2009) notes that democratic parties in Russia were not receptive to the idea of gender equality precisely at the time when these political parties were most successful in Russian politics. They began to show some interest in gender issues only to the extent that they themselves became marginalized (Zdravomyslova, 2009). Both politicians and intellectuals of that time were not ready to recognize gender inequality as a serious problem in Russia, penetrating the family, education, the economy and politics. Zdravomyslova (2009) adds that they were “far from understanding that raising the issues of achieving gender equality means speaking the language of modern democracy and discussing one of its key issues” (p. 2), which, in turn, gave an impetus to the processes of re-traditionalization and returning to “authentic” images in terms of gender norms (Ishkanian, 2005).

These political, economic and cultural challenges made many women in post-Soviet countries to unite under different non-governmental organizations (NGOs), which were fully or partly funded by Western donors such as MacArthur Foundation (Pushkareva & Bolshakova, 2020). Funding from Western donors was also provided for summer courses, curriculum development, support for women’s academic publications, websites, international women’s organizations, and grants for participation in international conferences (Chatterjee & Petrone, 2019). Many of these women’s NGOs in post-Soviet countries were founded first as charity organisations to compensate for the lack of adequate social services helping women to solve their personal and family problems through their activism and leadership roles in NGOs (Ahmedshina, 2007; Phillips, 2000). For example, in 2002, there were about 400 women’s organizations in Russia. Owing to their participation in NGOs, women gained knowledge and skills in gender activism and expanded their network of gender advocates and scholars.

Similar to Russia, women’s NGOs were the pivot around which women activists mobilized themselves in other post-Soviet contexts. For example, around 100 women’s NGOs were created in the Republic of Uzbekistan during the years of independence such as the Committee of Women of Uzbekistan, Association of Business Women of Uzbekistan, “Tadbirkor Ayol” and Republican Association of Women Scholars, “Olima”. Similar to any other post-Soviet country Uzbek women saw NGOs as an opportunity and a tool that helped them to express their problems. The activities of women’s NGOs, supported and financed by international agencies and organizations, undoubtedly contributed to the spread of the idea of gender equality and the recognition of the important role of women’s organizations in the construction of civil society (Ahmedshina, 2007). Related processes have been observed in Ukraine, although several competing discourses circulated within NGOs in post-Soviet Ukraine (Philips, 2000). If sometimes the motherly role of women was glorified and women’s values were romanticised, the feminist discourses encouraged women to re-think traditional gender roles and call for their social and political activeness (Phillips, 2000). Likewise, in post-Soviet Turkmenistan, community centers became the key site for gender activism. In 1998 women’s community centers were created on the initiative of the Women’s Union of Turkmenistan that have become fundamental institutions to develop and implement the projects of women’s movement in the country (Almamedova, 2011). Although NGOs’ activities were aimed at challenging the traditional social norms and gender stereotypes that restrict women’s creative potential, NGOs operated only in large cities ignoring the rural women whose economic, political and public life was negatively affected by the transition period (Ahmedshina, 2007).

Among these non-governmental organizations is the famous Kharkiv Center of Gender Studies, established in 1992 by a group of teachers, graduate students and students of Kharkiv universities to popularize and introduce gender and women’s studies into the system of post-Soviet higher education. In the 1990s and early 2000s, the center’s participants, in collaboration with colleagues from various universities in Ukraine, prepared many training courses and modules, which are still taught at universities in Ukraine and the countries of the former Soviet Union, as well as various educational materials, including the first post-Soviet textbook *Th*e*ory and A History of Feminism* published in 1996 and translated into Armenian and Kazakh languages. Since the beginning of the 2000s, the center has worked on the institutionalization of gender studies in post-Soviet universities and the training of specialists in the field of gender studies in the countries of the former Soviet Union and research and publishing work in various areas of gender studies (Bezrukova, 2011). Irina Zherebkina, as the director of the Kharkiv Center for Gender Studies, founded the first networked academic project on gender studies in the former Soviet Union, namely *the University Network on Gender Studies for the Countries of the Former USSR* (Zherebkina, 2003). The center publishes the famous scientific journal *Gendernie Issledovaniya (Gender Studies)*, a popular feminist publication not only in Ukraine, but throughout the post-Soviet space. Zherebkina (2003) also notes that among non-university centers for gender studies is the St. Petersburg Center for Gender Problems. She adds that most of these non-university centers distinguish themselves from conservative established universities. Paradoxically, many women connected with these centers were professors of those universities. Furthermore, Grishak’s (2018) analysis of the theoretical sources on gender studies confirms the idea that gender curriculum in higher education in Central Asian countries did not arise in the academic environment of universities, but instead, it was initiated by female researchers and activists of women’s NGOs, who, with the support of foreign organizations and funds, were involved in gender studies.

Khotkina (2020) argues that the development of gender studies in Russia took place in three stages. The first period was labelled as “gendered 90 s” indicating the intensive beginning of gender studies which led to the opening of the Laboratory of Gender Studies at the Institute for Socio-Economic Problems of Population (ISEPN) under the Russian Academy of Sciences in 1990 mentioned earlier. The establishment of this laboratory meant that gender studies were, to some extent, recognized and legitimized by the Russian Academy of Science. However, one of the important characteristics of this stage was the first step towards establishing closer contacts and connections between gender researchers from Russia and the post-Soviet countries. In January 1996, a scientific conference entitled Gender Studies in Russia: Problems of Interaction and Prospects for Development allowed the first meeting of Russian scholars to discuss institutional, methodological, social and other issues related to gender/women’s studies and their teaching in Russian higher education. This initial step resulted in an information network that brought together gender scientists and teachers from Russia and other post-Soviet countries. In addition, the scholar based at Ivanovo State University created educational programmes and established the interuniversity scientific center on gender and established the scientific journal “Women in Russian Society” that aimed to increase theoretical and methodological publications in gender studies and to disseminate the ideas on ways to achieve gender equality among the general public.

In contrast to the 1990s, the second stage fell in the first decade of the twenty-first century (2000–2009), where the ideological and political priority was not about democratization but the stability and consolidation of the new political power in Russia. In this period, the themes of demographic threats and consolidation of traditional family values dominated gender research (Zdravomyslova, 2009). However, some of the most significant Russian scientific schools involved in gender studies such as the European University in St. Petersburg and St. Petersburg State University were founded in the 2000s. These can be characterized as having a sociological orientation if one looks at the priority of research and publications produced by the two organizations (Khotkina, 2020). Other examples include Moscow Center for Gender Research and Moscow State University, and the Center in Saratov. All of these paid the greatest attention to research and analysis of socio-economic gender disparities in Russian society (Khotkina, 2020).

The third stage in the development of gender studies in Russia began after 2010 due to the adoption of conservative laws and the mobilization of conservative movements. The third stage is marked by the fact that in the context of the spread of information and communication technologies (ICT), the methods and speed of obtaining and distributing information have radically changed, as well as new information resources have appeared in the form of social networks, Internet forums and blogs on gender studies (Chemankova & Hen, 2019; Khotkina, 2020).

One of the key features of the development of gender studies in post-Soviet space is that they go simultaneously with nation-building processes (Zhurzhenko, 2008). Therefore, the ideology of retraditionalisation and focus on the family is common in many post-Soviet countries. Rezvushkina’s (2020) research demonstrated the role of mother-women in the national state revival in modern Kazakhstan. Moreover, not all researchers see the “problem of gender” as a problem of power relations (Pershai, 2013; Zhurzhenko, 2008). As Pershai (2013) argued, usually, the social and political conceptualization of gender is replaced by the introduction of individual “forgotten” or “lost” women into historiography. This approach, in fact, reproduces the existing patriarchal cultural norms locating the “women’s” theme only in its traditional form without performing critical gender analysis (Pershai, 2013). Post-Soviet space, according to Zherebkina (2003), also implements a version of gender studies where the Ministry of Education in Ukraine requires universities to introduce mandatory gender studies into the curricula. An example is PROUN project in Ukraine; however, at the same time, such actions are built and exercised by power relationships of male rectors and male editors of journals published by these higher education institutions (Zherebkina, 2003). She also notes that ‘the paradox afflicting the common strategies of post-Soviet gender institutionalization is that these kinds of projects are likely not only to survive but also sanction the new social discipline..’ (Zherebkina, 2003, p. 73), such as gender studies in the post-Soviet space.

On the whole, most of these non-governmental centers and higher education institutions in post-Soviet countries publish their own journals dedicated to gender and women studies; however, many of these journals are not part of large databases such as Scopus or Web of Science, including the studies in this literature review. For example, the leading journal of *Gendernie Issledovalniya* (Gender Studies) issued by Kharkiv Center of Gender Research is open access and “does not share the policies of academic publishers and information resources, seeking to control the publications of scientists worldwide and selling access to their research through a system of copyrights and subscriptions” (http://kcgs.net.ua/journal-gs.html).

Meanwhile, the breakup of the Soviet Union unlocked the beginning of the transformation of post-Soviet science; however, this transformation was accompanied by the financial crisis and scarce resources for research and development in post-Soviet countries (Hernandez-Torrano et al., 2021; Heyneman, 2010; Johnson, 2008). Thus, the research activity in the first years of independence in the 1990s was minimal. At the beginning of the 2000s, the post-Soviet countries attempted to transform their policies regarding research and science. The initiatives included integrating research and science into the higher education systems as opposed to the Soviet time when the research was primarily placed in the Academy of Sciences, and the universities were considered as teaching institutions (Kataeva & DeYoung, 2018). For instance, the establishment of Excellence Initiatives in Russia and flagship universities such as the University of Tartu in Estonia and Nazarbayev University in Kazakhstan aimed to contribute to the development of research and science in their respective countries and in the region (Tamtik & Sabzalieva, 2018).

For the past years, the interest in different patterns of publications and research in post-Soviet countries has increased significantly. Several publications attempted to examine research in various academic fields in post-Soviet countries. For example, Chankseliani (2017) analyzed international and comparative education research articles to chart the development of Soviet and post-Soviet education knowledge through three comparative and international education journals. She found that scholarship's thematic, theoretical, discursive, and methodological aspects are linked with changing geopolitical realities. Lovakov and Agadullina (2019) analyzed publications from post-Soviet countries in psychological journals. They found that from 1992 to 2017, 15 post-Soviet countries published less than one percent of the world output in psychological journals. Nevertheless, the publication number experienced growth, especially in Russia and Estonia. Lovakov and Yudkevich (2020) studied the articles on higher education published in academic journals in the last three decades. They found that only Russia, Lithuania, and Estonia have a larger publication output with more than 100 articles in journals indexed in Scopus, compared to other post-Soviet states. Chankseliani et al. (2021) conducted a bibliometric study on the quantity and impact of academic publications and the quality of journals in which these scholarly articles are published. The study’s findings demonstrated that all fifteen post-Soviet countries approach research differently, and the quantity, quality, and impact of their publications contribute less to global research output. Hernandez-Torrano et al. (2021) sought to investigate the development and structure of post-Soviet education research since the collapse of the Soviet Union. Their analysis showed an increase in education research in post-Soviet countries; however, the contribution of post-Soviet education research to international literature remains marginal. More recently, Matveeva et al.’s (2022) review of international scientific collaboration found that patterns of international cooperation in post-Soviet countries have significantly changed over the past decade, moving from intra- to inter-country collaboration. Changes have also been observed in the latter, with collaboration between post-Soviet countries going down and partnerships with Western countries going up.

While the preceding studies sought to understand different aspects of post-Soviet research and publications, none of these studies focused on the development and structure of gender studies, including publication output, leading countries in gender research publications, institutions and authors, and patterns of scientific collaboration. The current bibliometric study seeks to address this gap.

## Methodology

Recently, bibliometric research analysis has increased considerably in various domains and disciplines. Bibliometric analysis is a systematic method to understand and determine research trends. With the rapid development of data mining and graphics visualization technologies, it becomes possible to use software programs to analyze large amounts of data and visualize results. For this study, we used a bibliometric approach to map the literature on gender research in all 15 post-Soviet countries over the last 30 years. We used both performance analysis and science mapping. Performance analysis examined the contributions of researchers in the field of gender. We used the most prominent performance analysis measures, such as the number of publications and citations in gender since 1993. Science mapping was used to analyze the relationship between researchers, namely intellectual interactions and structural collaborations among researchers. This technique included citation analysis, collaboration analysis, co-authorship analysis, keyword analysis, and topical foci analysis. The WoS database was selected for analysis because it is the largest database covering over 76 million records and over 1600 million cited references. Furthermore, this database includes influential studies in scientometric and bibliometric research published in primary academic journals worldwide.

### Search strategy

**Fig. 1:**
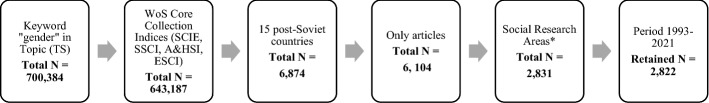
Search strategy. *Psychology, Education Educational Research, Business Economics, Sociology, Social Sciences Other Topics, Arts Humanities Other Topics, History, Linguistics, Philosophy, Government Law, Area Studies, Anthropology, Women Studies, Social Issues, Family Studies, Behavioral Sciences, Communication, Literature, Social Work, Demography, Religion, International Relations, Public Administration, Art, Information Science Library Science, Ethnic Studies, Development Studies, Urban Studies, Cultural Studies, History, Philosophy of Science, Asian Studies, and Music

**Fig. 2:**
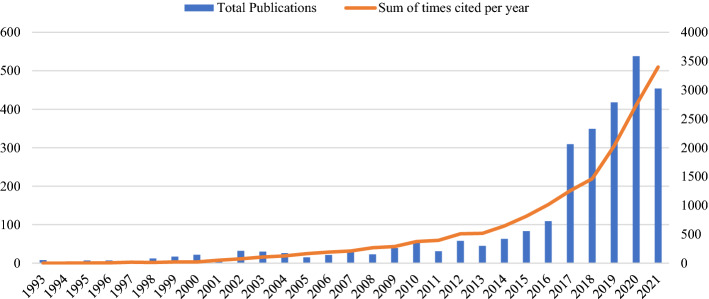
Distributions of gender research publications between 1993 and 2021 in post-Soviet countries. (Color figure online)

The entire search and selection process took place in January 2022. Figure 1 demonstrates the search strategy and procedures performed in filtering the metadata. Our search strategy was based on publications where the keyword “gender” appeared in the title, abstract, or author keywords. The second step consisted of refining the obtained results and filtering publications that were not relevant. The results were refined based on four indexes: Science Citation Index—Expanded (SCIE), Social Sciences Citation Index (SSCI), Arts and Humanities Citation Index (A&HCI), and the Emerging Science Citation Index (ESCI). The results were also filtered by 15 post-Soviet countries (Armenia, Azerbaijan, Belarus, Estonia, Georgia, Kazakhstan, Kyrgyzstan, Latvia, Lithuania, Moldova, Russia, Tajikistan, Turkmenistan, Ukraine, and Uzbekistan) and the document type, as we focused only on articles. We excluded review articles for two reasons. First, articles report original research and review articles do not present any new information on the research topic. Second, it allowed us to create an effective, focused overview of existing research. We also filtered the research areas, and all the authors discussed which research areas should be included as we focused on social science. We also applied a time frame of the period since the collapse of the Soviet Union in 1991. There were no publications in the first two years (1991 and 1992), so the results include the period from 1993 to 2021 (December). Finally, after refining the documents, a final sample consisting of 2822 publications and bibliometric metadata was extracted to analyze different types of figures and tables. Since the growth of gender research publications has remarkably increased between 2017 and 2021, we have conducted additional analysis to capture the nuances of gender research evolution in post-Soviet countries in these four years (Fig. 2).

### Data analysis

The data pertaining to the study has been retrieved from the WoS database and analyzed using VOSViewer software version 1.6.11 and MS Excel. These programs enable examining the dynamics and structure of the data extracted. VOSviewer is a software developed by Van Eck and Waltman (2017) from Leiden University in the Netherlands. The software is designed to create and visualize econometric networks for countries, organizations, journals, researchers, keywords, and publications based on co-citation, coupling, and co-authoring relationships. In addition, VOSviewer is a free software that allows the creation and visualization of maps based on relationships between existing items (www.vosviewer.com). In this study, we used the number of links and total link strength to calculate the significance of a given article in a specific cluster. A link is a relation between two publications in bibliographic coupling analysis, and each link is assigned a strength. Van Eck and Waltman (2018) programmed VOSviewer to calculate the strength values and demonstrate a total link strength.

### Limitations of the study

The bibliometric analysis based on the databases like WoS or Scopus does not allow us to capture the whole development of gender research in post-Soviet countries. As we mentioned earlier, most of the articles on gender studies in the post-Soviet area are published outside international journals and not indexed by international research databases like WoS. Numerous local scholars and institutions in gender studies publish mainly in local journals. For example, “Gendernye Issledovania” (Gender Studies), published by the Kharkiv Center for Gender Studies since 1998 and one of the leading journals on gender studies in the post-Soviet space is indexed neither by the Web of Science Core Collection nor by Scopus.

## Results and discussion

### Growth of publications

This study found 2822 publications on gender in post-Soviet countries in the WoS database between 1993 and 2021, representing only 0.4% of all gender-related publications in the WoS Core Collection. The 2822 publications in the dataset were published by 7370 unique scholars affiliated with 2685 institutions in 121 different countries worldwide. This accounts for all players producing gender research in post-Soviet countries and their collaborators in non-post-Soviet countries. Our findings indicate that the majority of publications were published in English (1695; 60.1%) and Russian (1033; 36.6%). Other encountered languages included Ukrainian (27; 0.9%) and Lithuanian (23; 0.8%). The small percentage of gender research in post-Soviet countries suggests that the contribution of these states to international gender research literature since the collapse of the Soviet Union remains insignificant.

Figure 2 presents the annual publications and the citation structure of the publications published each year. The results of our analysis suggest that the publication rate in gender research remained very low for the first two decades after the collapse of the Soviet Union and dramatically increased after 2016. Upon closer examination, we determined that only 26.7% were published in the first 24 years, and the remaining 73.3% of all publications were published in the last 5-year period (Fig. 2). Hernandez-Torrano et al. (2021) suggest that this growth likely points to the Web of Sciences Emerging Science Citation Index (ESCI) launch in late 2015 rather than an increase in actual publications. Furthermore, to increase the research activity of universities in many post-Soviet countries, higher education institutions started adopting the new policy of publication in high impact factor journals. Universities started counting the research productivity of scholars and faculty members by the number of publications in international journals indexed by the databases like WoS and Scopus, which are also ranked in the first and second ranking (quartiles). The number of articles published in such journals started influencing the scholars’ wages and funding for research institutions. One such example is the National Research University of Higher School of Economics in Russia, which is a participant of the 5–100 Excellence initiated by the government. Thus, we assume that the increased visibility of gender research in post-Soviet countries in the last ten years corresponds to the inclusion of local journals into ESCI rather than publications in international journals, while English remains the dominant language following Russian.

### Prolific countries, institutions, and authorship pattern in post-Soviet gender research

Before proceeding to the next stage of the analysis, it is worth mentioning that the territory of the former Soviet Union included both Asian and European continents, including Central Asia, the Far East, and the Baltic states. All 15 countries differed by size, population, languages, cultures, and economic indicators. Russia was the largest country within the Soviet Union, with the capital of Moscow, while some countries located in the Caucasus and Central Asia, such as Armenia, Tajikistan, and Kyrgyzstan, were the smallest. This difference is observed in total publications output as well. Table 1 presents the total number of publications and citations in gender research of the 15 post-Soviet countries and the ratio of publications per million inhabitants. The results showed significant differences between the post-Soviet countries regarding their input to gender research. According to the data, Russia and Estonia have the most considerable number of publications per million, and Central Asian countries have fewer research publications. Accordingly, Russia is leading with 61.4% of total input in the generation of gender research, followed by Estonia (10.8%), Ukraine (8.8%), and Lithuania (8.5%).

**Table 1:** Citation structure of gender publications in post-Soviet countries

Rank	Country	TP	TC	Inhabitants*, 2020	TP/million inhabitants	TLS
1	Russia	1758	6814	144,104,080	12.20	719
2	Estonia	310	5653	1,331,057	232.90	633
3	Ukraine	253	745	44,134,693	5.73	69
4	Lithuania	243	2053	2,794,700	86.95	190
5	Kazakhstan	84	319	18,754,440	4.48	56
6	Latvia	51	246	1,901,548	26.82	8
7	Georgia	45	284	3,714,000	12.12	23
8	Azerbaijan	34	114	10,110,116	3.36	10
9	Belarus	22	91	9,398,861	2.34	2
10	Kyrgyzstan	26	196	6,591,600	3.94	8
11	Uzbekistan	13	24	34,232,050	0.38	7
12	Armenia	10	26	2,963,234	3.37	11
13	Moldova	5	1	2,617,820	1.91	0
14	Tajikistan	6	60	9,537,642	0.63	2
15	Turkmenistan	1	3	6,031,187	0.17	0

*TP* total publications, *TC* total citations, *TLS* total link strength

*Data on country population were obtained for 2020 from https://data.worldbank.org/

However, to capture the nuances of gender research evolution and its significant growth, we conducted a deeper analysis of citation structure of gender publications between 2017 and 2021 (Table 2). The scrutiny of the data showed a remarkable change in total publications output by countries. We observed that Ukraine has significantly improved its position among post-Soviet countries in the last four years, positioning itself ahead of Estonia.

**Table 2:** Citation structure of gender publications in post-Soviet countries in 2017–2021

Rank	Country	TP	TC
1	Russia	1362	1929
2	Ukraine	228	319
3	Estonia	146	1021
4	Lithuania	145	439
5	Kazakhstan	76	245
6	Latvia	38	105
7	Azerbaijan	29	70
8	Georgia	28	124
9	Kyrgyzstan	19	56
10	Belarus	17	69
11	Uzbekistan	9	7
12	Armenia	8	26
13	Moldova	4	1
14	Tajikistan	3	0
15	Turkmenistan	0	0

*TP* total publications, *TC* total citations

In total, 7406 authors have participated in the publication of the papers included in the final sample giving a mean of 1.3 authors per document. Tables 3 and 4 demonstrate the top institutions, authors, and countries which published gender research papers from 1993 to 2021 in post-Soviet gender research. The tables include ranks, number of publications, total citations, and total link strength. According to our data, Estonian and Russian higher education institutions (the University of Tartu and National Research University Higher School of Economics) are leading in gender research publication output. Authors from Estonia, such as Tulviste from the University of Tartu, have been ranked first and contributed 21 publications with the highest total citations of 284. Puur and Kikas of Estonia were in the 2nd and 3rd positions from Tallinn University with 13 and 12 publications. The reason for Estonia as a country with the leading institutions and authors might be explained by the development of superior research policies and infrastructure after the collapse of the Soviet Union. Tamtik and Sabzalieva (2018) argue that including Estonia in the European Union was a determining factor in regaining the country’s legitimacy both internally and globally. Furthermore, Estonia was one of the first countries to sign the Bologna declaration and start large-scale higher education reforms. The University of Tartu, the first national university, followed the model of German and Swedish universities, becoming one of the leading institutions in the post-Soviet space. These findings are similar to the previous study conducted by Hernandez-Torrano et al. (2021) which found Estonia as a top producer of publications in education research.

**Table 3:** Leading institutions in gender research in post-Soviet countries

Rank	Institution	Country	TP	TC	TLS
1	University in Tartu	Estonia	189	3497	43
2	National Research University Higher School of Economics	Russia	132	343	48
3	Lomonosov Moscow State University	Russia	131	455	17
4	National Research University	Russia	115	625	53
5	Saint Petersburg State University	Russia	95	371	12
6	Tallinn University	Estonia	81	1305	69
7	Tomsk State University	Russia	57	266	10
8	Moscow State University Of Psychology And Education	Russia	53	111	15
9	Kazan Federal University	Russia	51	82	3
10	Vilnius University	Lithuania	44	225	8
11	Ural Federal University	Russia	41	68	1
12	Vytautas Magnus University	Lithuania	38	103	10
13	Nazarbayev University	Kazakhstan	36	88	4
14	Lithuanian Sports University	Lithuania	32	84	3
15	European University at Saint Petersburg	Russia	26	123	11

*TP* total publications, *TC* total citations, *TLS* total link strength

**Table 4:** Leading authors in gender research in post-Soviet countries

Rank	Author	Current affiliation	Country	TP	TC	TLS
1	Tulviste, T.	University of Tartu	Estonia	21	284	3
2	Puur, A.	Tallinn University	Estonia	13	195	70
3	Kikas, E.	Tallinn University	Estonia	12	287	3
4	Slobodskaya, H. R.	Research Institute of Neuroscience and Medicine	Russia	11	134	0
5	Realo, A.	University of Warwick	UK	10	857	3
6	Maslauskaite, A.	Vytautas Magnus University	Lithuania	9	41	19
6	Grigorenko, E. L.	University of Houston	USA	9	82	1
7	Almukhambetova, A.	Nazarbayev University	Kazakhstan	8	31	20
7	Kuzhabekova, A.	Nazarbayev University	Kazakhstan	8	36	19
7	Enikolopov, S. N.	Mental Health Research Center	Russia	8	40	0
7	Kim, E.	American University of Central Asia	Kyrgyzstan	8	13	0
7	Sergeeva, M. G.	Peoples' Friendship University of Russia	Russia	8	2	0
7	Tikhomirova, T. N.	Lomonosov Moscow State University	Russia	8	85	0
8	Rahnu, L.	University in Turku	Finland	7	79	58
8	Sakkeus, L.	Tallinn University	Estonia	7	86	53
8	Mottus, R.	University of Edinburgh	UK	7	363	3
8	Temkina, A.	European University at St. Petersburg	Russia	7	94	0
8	Welzel, C.	Leuphana University Lueneburg	Germany	7	126	0

*TP* total publications, *T* total citations, *TLS* ttal link strength

As expected, Russian universities lead gender research publication output with two prominent universities being the Higher School of Economics and Lomonosov State University. Although the Higher School of Economics is a relatively new university in Russia, the university built a profound research culture that allowed this university to be among the top-ranked universities in the country. Moreover, the Higher School of Economics, the Kazan Federal University, and the Ural Federal University are participants of the 5–100 Excellence Initiative started by the government that aimed to include at least five universities among the top 100 ranked universities globally. We also observe a growth of research by authors from Central Asian universities representing Kazakhstan, such as Nazarbayev University and an author from the American University of Central Asia located in Kyrgyzstan. Nevertheless, we observed a prevalence of prolific institutions producing gender publications in the post-Soviet space and authors from a few countries. Moreover, despite the growing number of publications by post-Soviet scholars, the most cited author appears to be in the United Kingdom, affiliated with the University of Warwick (Table 4). In addition, the same analysis between the period 2017 and 2021 revealed insignificant changes in terms of publication output by institutions. None of the Ukrainian higher education institutions were among the leading institutions in gender research, even though Ukraine has improved its overall rank between 2017 and 2021, suggesting that the country’s improved rank is not linked to a couple of high profile institutions. Instead, Ukraine’s enhanced productivity is a cumulative effect of productivity across several Ukrainian institutions.

### Research collaboration in post-Soviet gender research

**Fig. 3:**
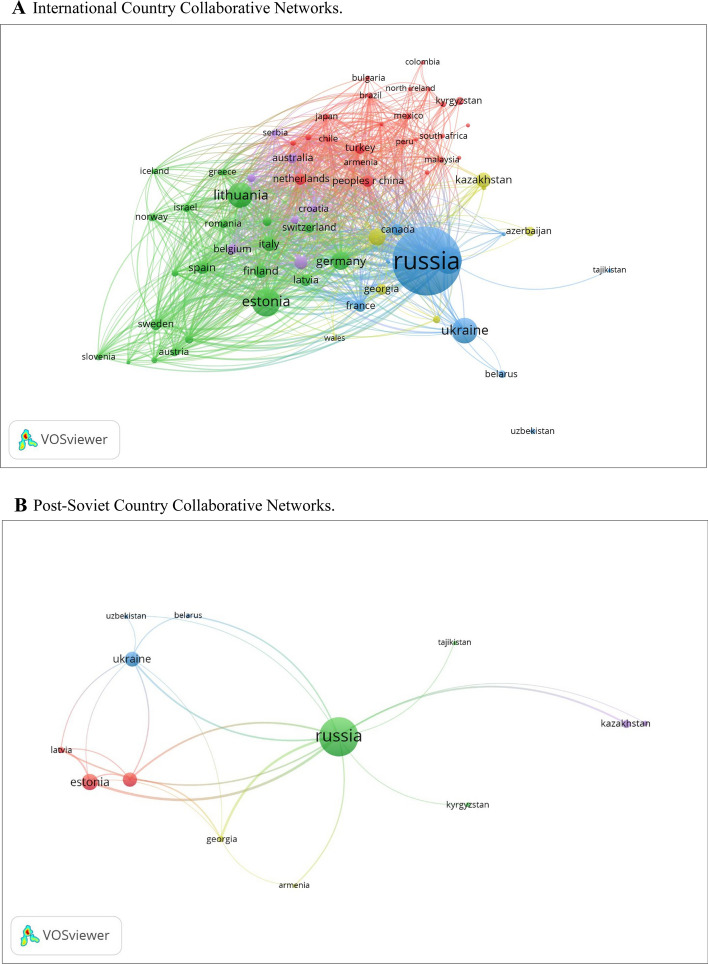

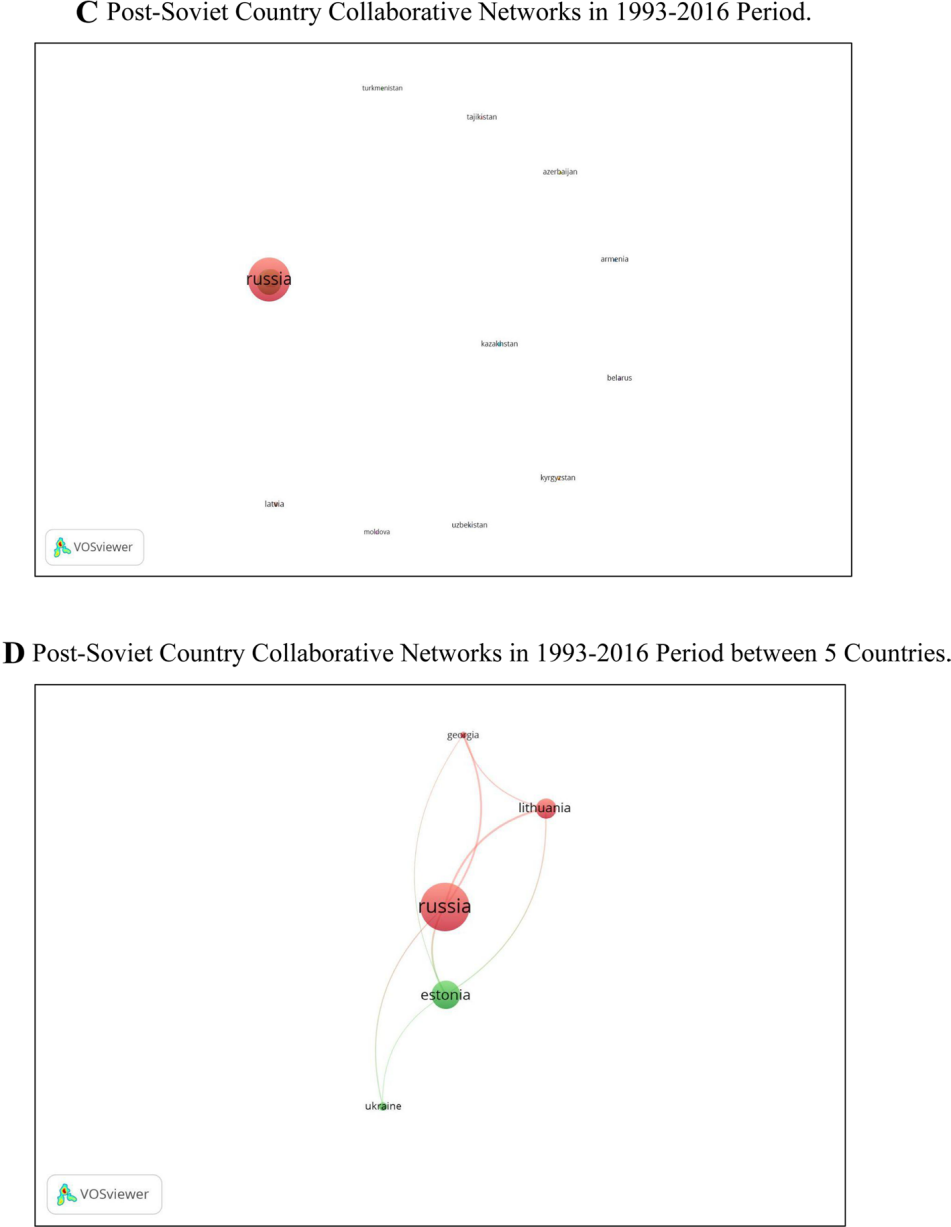

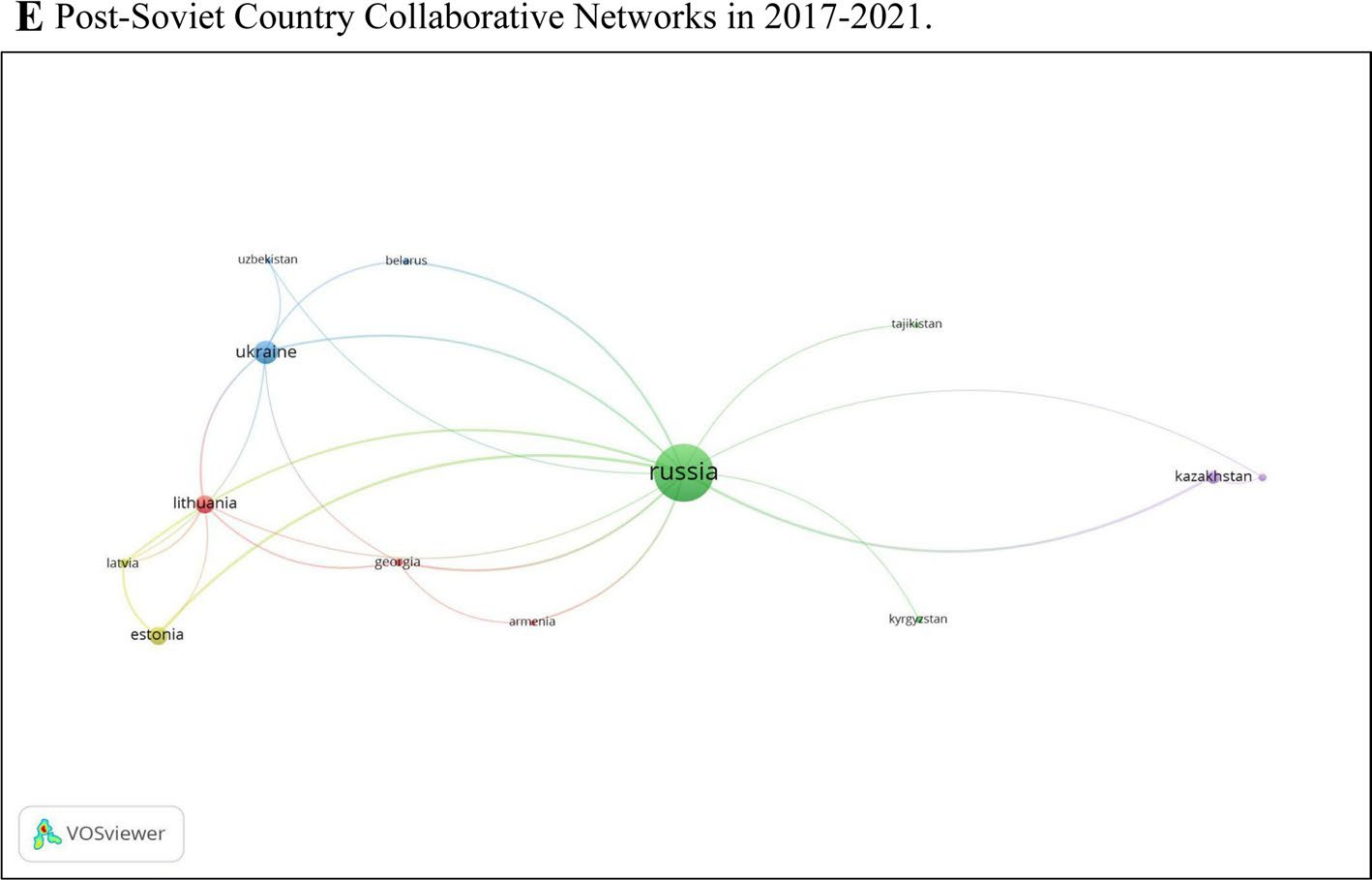
**A** International country collaborative networks. Co-authorship analysis for all countries with five or more publications in the dataset (n = 69). **B** Post-soviet country collaborative networks. Co-authorship analysis of post-Soviet countries with at least one publication in the dataset (n = 13). **C** Post-soviet country collaborative networks in 1993–2016 period. Co-authorship analysis of post-Soviet countries with at least one publication in the dataset (n = 15). **D** Post-soviet country collaborative networks in 1993–2016 period between 5 countries. Co-authorship analysis of post-Soviet countries with at least one publication in the dataset (n = 5). **E** Post-Soviet country collaborative networks in 2017–2021. Co-authorship analysis of post-Soviet countries with at least one publication in the dataset (n = 13)

The patterns of scientific collaboration in gender research in post-Soviet countries have risen over the past 30 years. The findings demonstrate the rise of co-authorship articles. Figure 3A displays the co-authorship analysis for all countries with more than five publications demonstrating international collaborations in the dataset (n = 69); 5 clusters show the scientific cooperation between countries. Our analysis indicates that researchers from Russia have solid scientific collaboration with researchers from Ukraine, Belarus, France, Canada, and Azerbaijan and the most negligible international collaboration with Central Asian countries. The other noticeable collaboration is between the Baltic states of Estonia, Lithuania, and Latvia. These post-Soviet countries mainly collaborate with European countries.

Figure 3B offers a visual representation of the scientific collaboration networks in the field when considering only the post-Soviet countries. The map shows three main clusters of cooperation and four secondary clusters, which are mainly determined by geographical proximity and economic unions. On the left, the Eastern European countries that are part of the European Union are grouped into a red cluster. The second blue cluster includes Belarus, Ukraine, and Uzbekistan. The third sizeable green cluster is represented by Russia, Kyrgyzstan, and Tajikistan. The rest of the countries show little ties with other countries in collaboration with gender publications.

Again, in order to capture the nuances of collaboration of gender research, we conducted a deeper analysis separating the periods between 1993–2016 and 2017–2021. Our analysis demonstrated weak collaboration between post-Soviet countries in the period 1993–2016 (Fig. 3C). In this period, researchers from Russia collaborated with Lithuania and Georgia, and Estonian researchers collaborated with Ukrainian colleagues (Fig. 3D), while the deeper analysis demonstrated a visible collaboration between post-Soviet countries after 2016 (Fig. 3E).

**Fig. 4:**
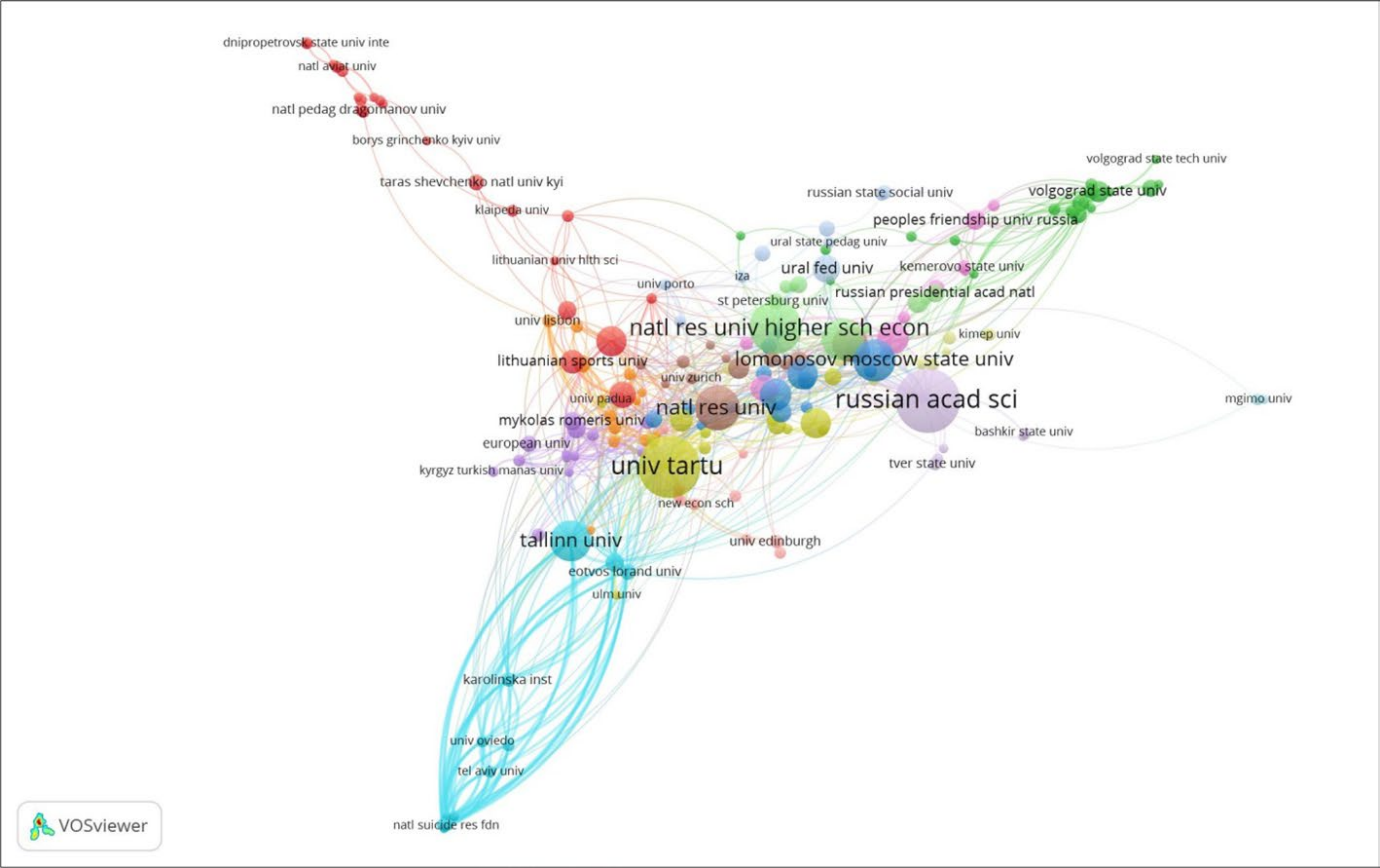
Affiliation collaborative networks in post-Soviet gender research. Co-authorship analysis of institutions with five or more publications in the dataset (*n* = 192)

Scientific collaborations among institutions with five or more publications are presented in Fig. 4 (n = 192). The clusters represent collaborations that are more frequent at the country level. These clusters suggest that institutions from Russia (Russian Academy of Science in violet), the Higher School of Economics (in light green), Moscow State University (in blue) and Estonian the University of Tartu (in yellow), and Tallinn University (in light blue) collaborate internally with very few collaborations with other post-Soviet countries and institutions.

**Fig. 5:**
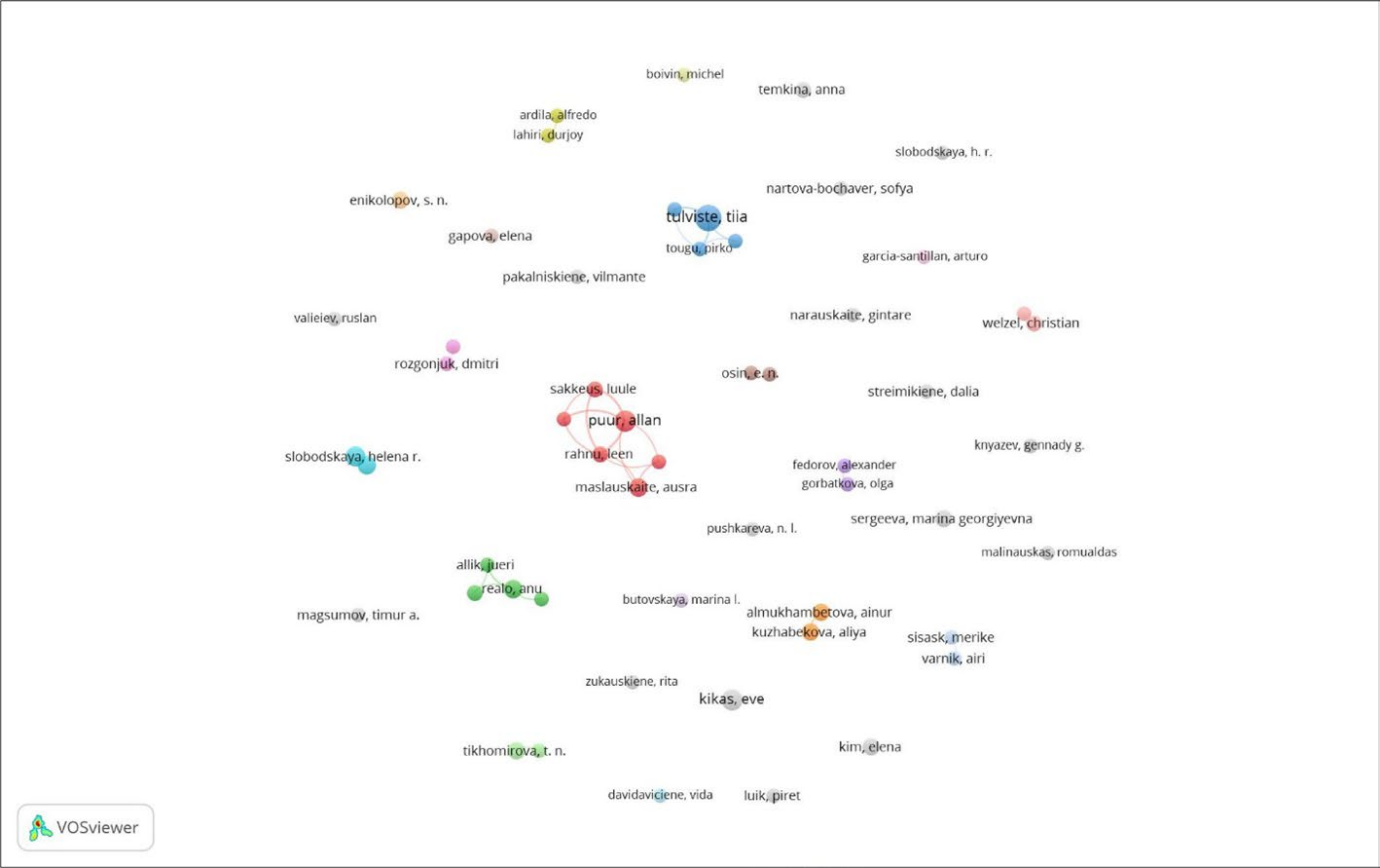
Author collaborative networks in post-Soviet gender research. Co-authorship analysis of authors with five or more publications in the dataset (*n* = 54)

Figure 5 demonstrates the co-authorship analysis for researchers with five or more publications (n = 54). The results suggest similar collaboration trends at the level of authors, where the authors collaborate mainly with other scientists associated with the same institutions or within the same countries. At the center of Fig. 5, we observe a cluster of researchers (in red) from Estonia’s Tallinn State University who strongly collaborate. Other clusters include co-authorships of Russian researchers and researchers from Kazakhstan (in orange and blue). This analysis shows that authors from post-Soviet countries mostly co-author papers with researchers from the same institutions and have almost no collaborations with other researchers from post-Soviet countries.

### Prolific journals

When it comes to the analysis of core journals, Table 5 demonstrates the leading ten journals in the field of gender. Journals with minimum productivity of 16 documents are listed. In total, 613 papers were published in the top 15 active journals, which accounted for 21.7% of the publications in the WoS core database. Journals published in Russia take the first six positions in journal rankings, with “Sotsiologicheskie Issledovaniya” (Sociological Studies) ranking first with 117 documents.

**Table 5:** Core journals in post-Soviet gender research

Rank	Journal	TP	TC	Country
1	Sotsiologicheskie Issledovaniya	117	138	Russia
2	Psikhologicheskii Zhurnal	83	289	Russia
3	Voprosy Psikhologii	68	110	Russia
4	Psychology in Russia-State of the Art	33	60	Russia
5	Vestnik Tomskogo Gosudarstvennogo	32	5	Russia
6	NAUCHNYI dialog	31	11	Russia
7	Frontiers in Psychology	26	198	Switzerland
8	Anthropological Measurements of Philosophical Research	25	14	Ukraine
9	Psychology and Law	24	15	Russia
10	Sibirskiy Psikhologicheskiy Zhurnal-Siberian Journal of Psychology	22	13	Russia
11	European Journal of Contemporary Education	21	24	Slovakia
12	Social Psychology and Society	20	15	Russia
12	Psikhologicheskaya Nauka i Obrazovanie-Psychological Science and	20	32	Russia
12	Journal of Social Policy Studies	20	14	Russia
13	Amazonia Investiga	19	28	Colombia
13	Suicidology	19	15	Russia
14	Vestnik Volgogradskogo Gosudarstvennogo Universiteta-Seriya	17	7	Russia
15	Sibirskii Filologicheskii Zhurnal	16	2	Russia

*TP* total publications, *TC* total citations

**Fig. 6:**
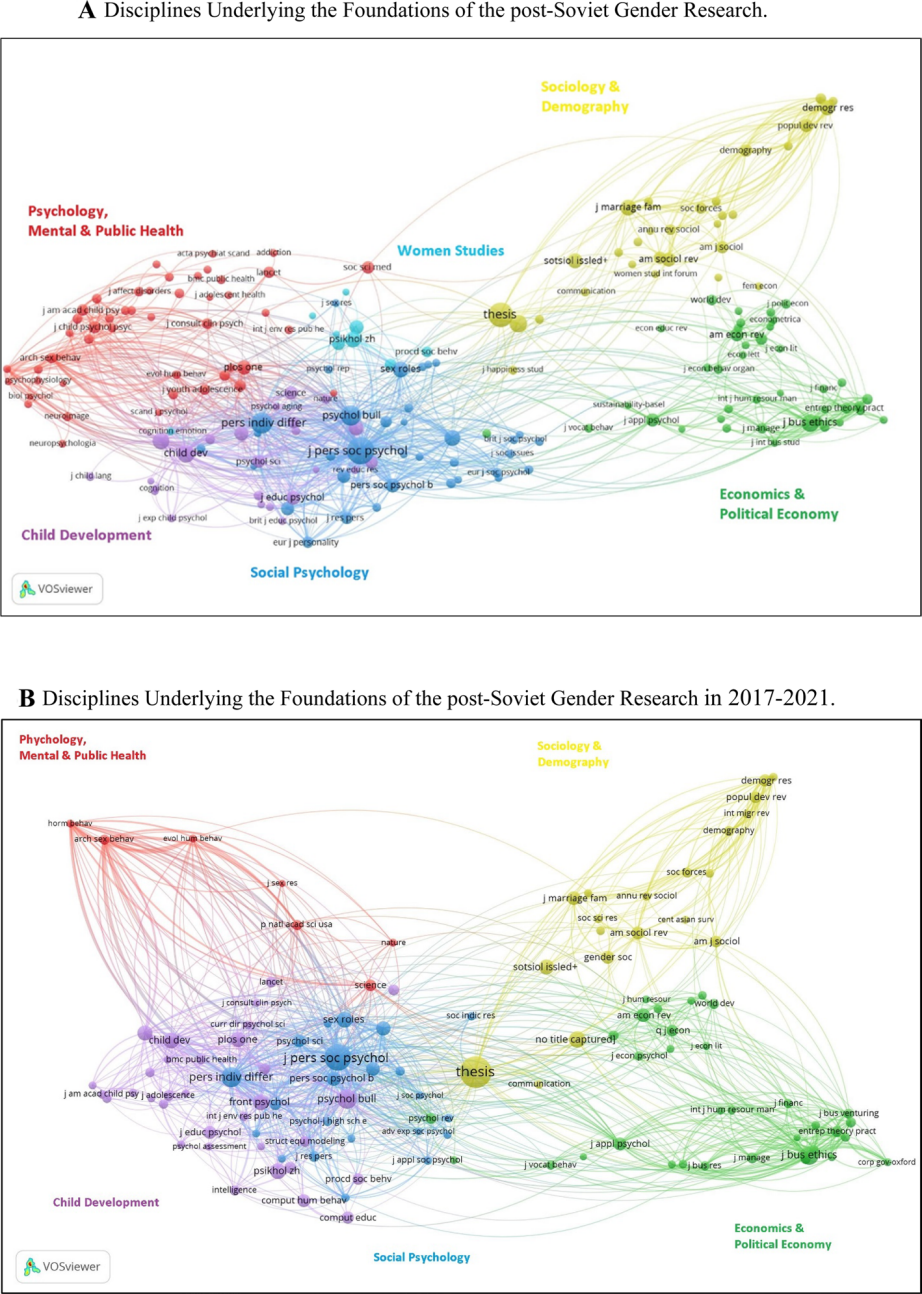
**A** Disciplines underlying the foundations of post-Soviet gender research. Co-citation analysis of journals with 50 or more citations in the dataset (*n* = 188). **B** Disciplines underlying the foundations of the post-Soviet gender research in 2017–2021. Co-citation analysis of journals with 50 or more citations in the dataset (*n* = 119)

Figure 6A demonstrates the co-citation analysis of journals with 50 or more citations. The map illustrates gender research in post-Soviet countries is based on the knowledge generated in six interrelated disciplines. The four clusters appearing together are highly interconnected with each other and related to journals in social psychology (blue), children development (violet), economics and political economy (green), and women studies (light blue). On the other hand, journals related to sociology and demography (yellow) and economics and political economy (green) are separated from the first four clusters.

A deeper analysis of the 2017–2021 period showed insignificant changes in interrelated disciplines except for journals in psychology and mental and public health that are separate from other clusters (Fig. 6B).

Table 6 lists the ten most influential papers published in gender research journals by scholars in post-Soviet countries ranked by total citations. The leading publication is “Why can’t a man be more like a woman? Sex differences in Big Five personality traits across 55 cultures” (Schmitt et al., 2008). The publication reports on sex differences in personality traits between men and women. The most frequent topics in the most cited papers appear to be psychology-related topics. One noticeable pattern in the most important articles is the inclusion of only those post-Soviet states located in Europe, such as the Baltic States, Ukraine, and Moldova. It means that other countries of post-Soviet space are not represented in the papers that are most cited internationally.

**Table 6:** Most influential publications in post-Soviet gender research

Rank	Authors	Publications	TC
1	Schmitt (2008)	Why can’t a man be more like a woman? Sex differences in Big Five personality traits across 55 cultures	651
2	Swami (2010)	The Attractive Female Body Weight and Female Body Dissatisfaction in 26 Countries Across 10 World Regions: Results of the International Body Project I	330
3	Brunner (2014)	Life-time prevalence and psychosocial correlates of adolescent direct self-injurious behavior: A comparative study of findings in 11 European countries	211
4	Flanagan (1998)	Ties that Bind Correlates of Adolescents’ Civic Commitments in Seven Countries	194
5	Ivanova (2007)	The generalizability of the Youth Self-Report syndrome structure in 23 societies	188
6	Eriksson (2012)	Differences between girls and boys in emerging language skills: Evidence from 10 language communities	182
7	Pruitt (1995)	For love and money: Romance tourism in Jamaica	170
8	Rescorla (2007)	Epidemiological comparisons of problems and positive qualities reported by adolescents in 24 countries	165
9	Muravyev (2009)	Entrepreneurs’ gender and financial constraints: Evidence from international data	158
10	Menesini (2012)	Cyberbullying Definition Among Adolescents: A Comparison Across Six European Countries	155

*TC* total citations

### Bibliometric analysis of the keywords

The keyword analysis demonstrates the relationships between the most frequently used keywords by authors (AKW) and Keyword Plus (KWP) in the dataset using the co-occurrence of keywords. To create a representative figure, we limited the number of words to those that appeared at least 30 times. Table 7 shows the 20 most frequently occurring AKW and KWP in gender research in post-Soviet countries. The most frequently used keywords are “gender”, “Russia”, “gender differences”, “women”, ‘adolescent’’ and “education”.

**Table 7:** Most frequently occurring AKW and KWP in post-Soviet gender research

Rank	Author keyword	Occurrences	Rank	KeyWord plus	Occurrences
1	Gender	321	1	Gender	309
2	Russia	87	2	Gender-differences	144
3	Gender differences	70	3	Women	104
4	Women	46	4	Behavior	89
4	Adolescents	46	5	Children	72
5	Education	43	6	Performance	70
6	Masculinity	42	7	Attitudes	67
7	Culture	40	8	Sex-differences	66
7	Students	40	9	Impact	65
7	Gender equality	40	10	Personality	57
8	Personality	39	11	Model	56
9	Estonia	38	12	Age	52
10	Identity	37	13	Health	51
11	Feminism	36	14	Education	47
11	Gender identity	36	15	Students	41
11	Adolescence	36	15	Stress	41
12	Lithuania	35	16	Adolescents	40
13	Children	33	17	Perceptions	39
14	Family	31	18	Depression	37
15	Age	30	18	Work	37
15	Youth	30	19	Risk	34

The most frequently used words between 2017 and 2021 haven’t changed significantly with the exception of the word “students” (Table 8).

**Table 8:** Most frequently occurring AKW and KWP in post-Soviet gender research in 2017–2021

Rank	Author keyword	Occurrences	Rank	KeyWord plus	Occurrences
1	Gender	249	1	Gender	215
2	Russia	63	2	Gender-differences	98
3	Gender differences	47	3	Women	75
4	Women	39	4	Performance	52
5	Students	38	5	Behavior	51
6	Education	35	6	Impact	50
6	Gender equality	35	7	Attitudes	46
7	Identity	32	8	Personality	41
7	Adolescents	32	9	Health	40
8	Masculinity	31	10	Children	38
9	Culture	30	10	Model	38
10	Feminism	29	11	Age	34
10	Gender identity	29	11	Education	34
11	Adolescence	26	12	Sex-differences	32
11	Gender stereotypes	26	13	Students	31
12	Youth	24	14	Perceptions	30
13	Children	22	15	Stress	29
14	Family	20	16	Risk	24
14	Age	20	17	Work	23
14	Lithuania	20	17	Validation	23

**Fig. 7:**
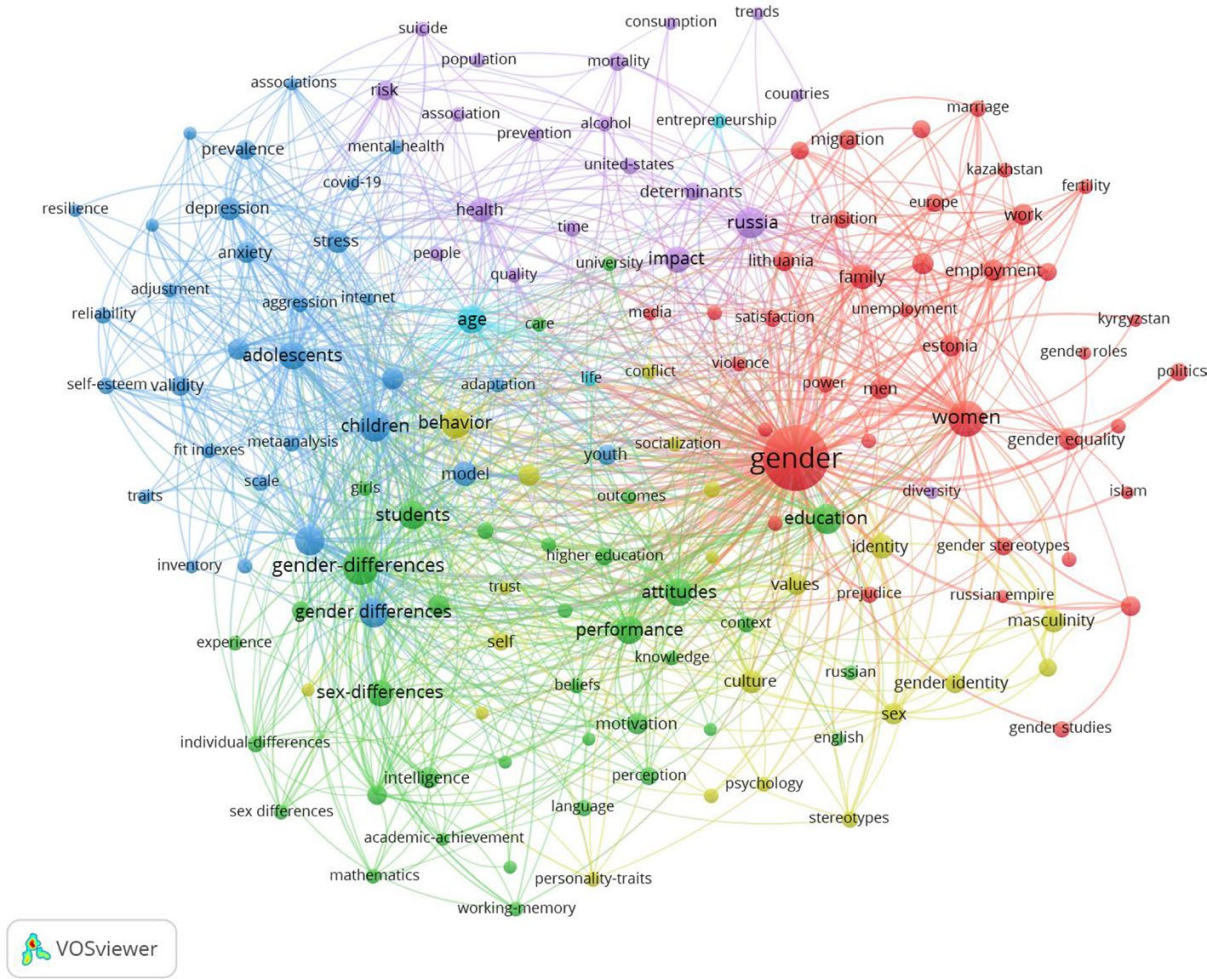
Topical foci of gender research in post-Soviet countries. Co-occurrence analysis of keywords occurring 15 or more times (*n* = 149)

Figure 7 presents the results of the co-occurrence analysis of all keywords in the dataset occurring 15 or more times (n = 149). The figure demonstrates the network map of the trend topics according to the keywords used by academics. The keywords included ‘gender’, ‘women’, ‘gender differences’, ‘sex differences’, ‘children,’ ‘students’, ‘children’s behaviour’, ‘education’, ‘attitudes’, and ‘performance’.

**Fig. 8:**
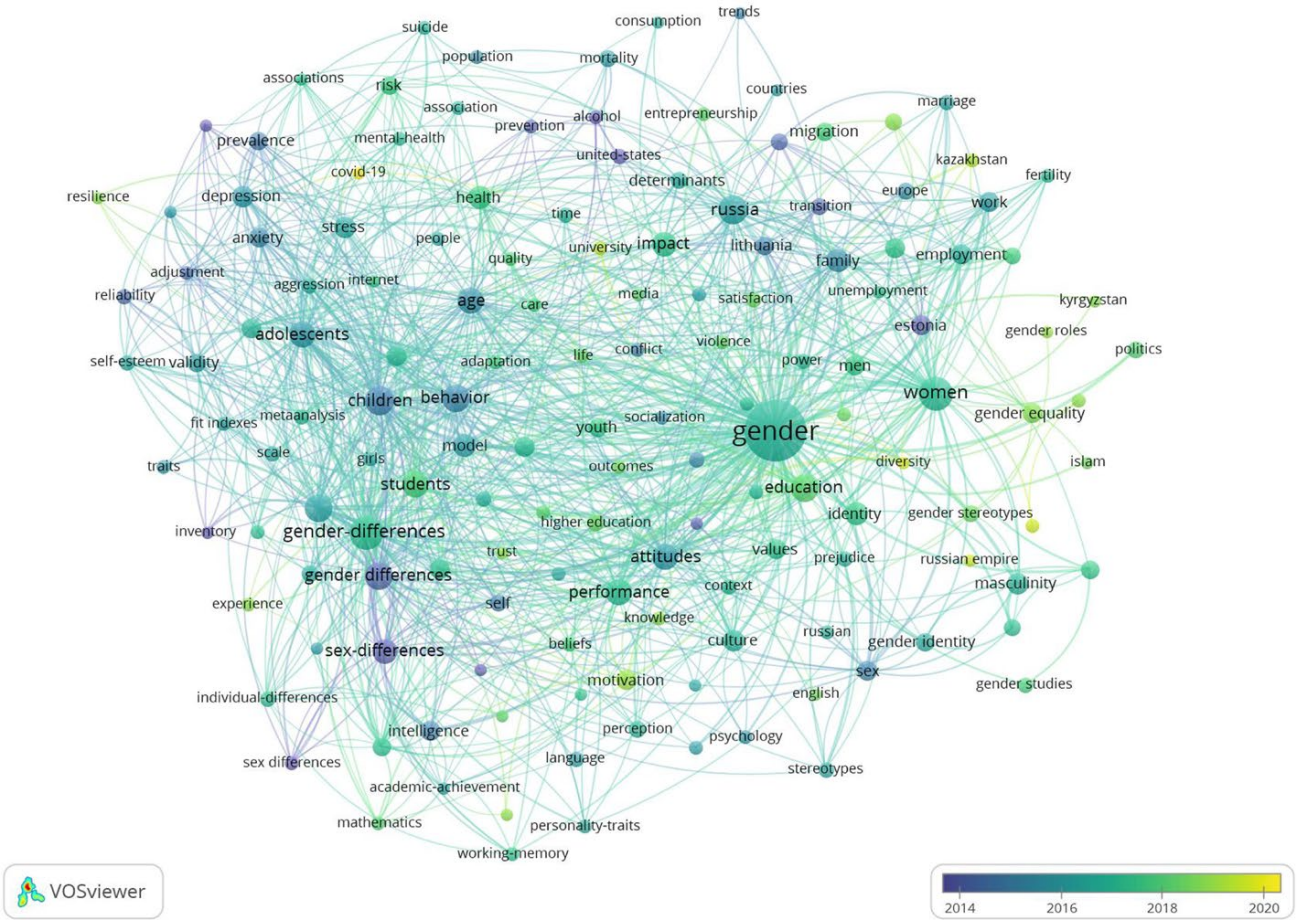
Interest evolution of gender research topics in post-Soviet countries. Co-occurrence analysis of keywords occurring 15 or more times (*n* = 149)

Figure 8 describes the evolution of the topical foci of gender research in post-Soviet countries. The figure shows the evolution of keywords over the studied period of 2015 to the end of 2021. While the main interest of academics in 2014 was about gender and sex differences among children and adolescents, over the last years, emerging topics demonstrated the transition of interest to gender, women, gender equality, and gender stereotypes, with the most recent topic of covid-19 (in yellow).

## Conclusion

Using bibliometric analysis, this study sought to understand the evolution of gender research in social sciences in post-Soviet countries since independence. The study included extract from 2822 publications indexed in the Web of Science Core Collection since 1993. The findings suggest that post-Soviet countries’ contribution to gender research remains insignificant, with articles primarily published in local rather than international journals. It should be noted that the vast majority of articles on gender are still published in journals not indexed by the Web of Science. Nevertheless, gender studies publications in the post-Soviet space have grown, particularly since 2017. There is unequal distribution among post-Soviet countries in terms of gender research publications. Russia and Estonia led the publication output between 1993 and 2016, while Ukraine moved from 3rd place in the same period to 2nd place between 2017 and 2021. The Caucasus and Central Asian countries, except Kazakhstan and Kyrgyzstan, contribute to gender research insignificantly. Estonia remains the leading country in publication relative to its population among other post-Soviet states. The variation in productivity might reflect the relative investment countries make in research and development since countries which invest more per researcher have a greater number of researchers which in turn leads to research productivity (Chankseliani et al., 2021). Although collaboration within countries and institutions is increasing, particularly between 2017 and 2021, researchers collaborate internally with colleagues from the same institutions. Most published work is related to psychology and behavioral sciences, looking at the biological differences between girls/boys and men/women; however, the analysis demonstrates that sociological and politically oriented gender research is evolving. The findings also suggest that topics of interest of post-Soviet researchers mostly align with global trends with the increase in sociological, demographic, and political economy disciplines, and there is a potential to contribute to international research in the future.
